# Factors Related to User Ratings and User Downloads of Mobile Apps for Maternal and Infant Health: Cross-Sectional Study

**DOI:** 10.2196/15663

**Published:** 2020-01-24

**Authors:** Rizwana Biviji, Joshua R Vest, Brian E Dixon, Theresa Cullen, Christopher A Harle

**Affiliations:** 1 Science of Healthcare Delivery College of Health Solutions Arizona State University Phoenix, AZ United States; 2 Department of Health Policy and Management Richard M Fairbanks School of Public Health Indiana University Indianapolis, IN United States; 3 Center for Biomedical Informatics Regenstrief Institute Indianapolis, IN United States; 4 Department of Epidemiology Richard M Fairbanks School of Public Health Indiana University Indianapolis, IN United States

**Keywords:** mHealth, mobile apps, pregnancy, parturition, infant care, smartphones

## Abstract

**Background:**

Mobile health apps related to maternal and infant health (MIH) are prevalent and frequently used. Some of these apps are extremely popular and have been downloaded over 5 million times. However, the understanding of user behavior and user adoption of these apps based on consumer preferences for different app features and categories is limited.

**Objective:**

This study aimed to examine the relationship between MIH app characteristics and users’ perceived satisfaction and intent to use.

**Methods:**

The associations between app characteristics, ratings, and downloads were assessed in a sample of MIH apps designed to provide health education or decision-making support to pregnant women or parents and caregivers of infants. Multivariable linear regression was used to assess the relationship between app characteristics and user ratings, and ordinal logistic regression was used to assess the relationship between app characteristics and user downloads.

**Results:**

The analyses of user ratings and downloads included 421 and 213 apps, respectively. The average user rating was 3.79 out of 5. Compared with the Apple App Store, the Google Play Store was associated with high user ratings (beta=.33; *P*=.005). Apps with higher standardized user ratings (beta=.80; *P*<.001), in-app purchases (beta=1.12; *P*=.002), and in-app advertisements (beta=.64; *P*=.02) were more frequently downloaded. Having a health care organization developer as part of the development team was neither associated with user ratings (beta=−.20; *P*=.06) nor downloads (beta=−.14; *P*=.63).

**Conclusions:**

A majority of MIH apps are developed by non–health care organizations, which could raise concern about the accuracy and trustworthiness of in-app information. These findings could benefit app developers in designing better apps and could help inform marketing and development strategies. Further work is needed to evaluate the clinical accuracy of information provided within the apps.

## Introduction

Increasingly, users are turning to digital technologies, such as Web-based and mobile platforms, where information regarding any topic is obtained at the touch of a button. There have been significant changes in the types of digital technologies that are available for use, and smartphones are increasingly the most popular devices for *on the go* information access [1,2]. Globally, there were 8 billion mobile-connected devices in 2016, and this number is estimated to grow to 11.6 billion by 2021 [3]. In the United States alone, 96% of adults owned a mobile phone in 2019, out of which 81% owned smartphones [2]. Today, smartphones are used for more than the basic features of calling, texting, or even browsing the internet. Users are using these devices to seek information on a wide range of life events, including their health [4]. This cultural shift has resulted in an increased access to health-related information for laypeople and has offered them a platform to engage in behavior modification activities [1]. Smartphones are popular for their abilities to support third-party programs, commonly known as mobile apps [5]. Since their first appearance in 2008, millions of mobile apps have been designed and published for smartphones, computer tablets, and other handheld devices [6].

The ubiquity of mobile phones offers a unique opportunity to use mobile health (mHealth) for health information seeking [7]. Recently, mHealth apps have gained popularity in providing pregnancy information with easy access at little or no cost, and women are increasingly using these platforms to meet information needs during pregnancy [8-11]. A large number of surveyed pregnant women and new mothers reported the use of such apps, with nearly a quarter using these apps almost daily [12]. A majority of first-time mothers and nearly half of experienced mothers found pregnancy and childbirth apps useful in providing valuable information [13]. In addition, these apps were deemed more useful by socially disadvantaged women who may otherwise lack access to alternate educational resources [8,14].

Compared with other health topics, mobile apps for maternal and infant health (MIH) subjects, such as pregnancy, childbirth, and infant care, are some of the most frequently developed and commonly used [9,10]. MIH apps often appear on the iTunes and Google Play Store’s list of most downloaded apps, and some of the apps have been downloaded over 5 million times [1]. Some of these apps have an average user rating (ie, *stars*) of 4.5 (out of 5), with higher ratings indicating a more favorable user experience. Both user downloads and user ratings offer an arbitrary indicator of the popularity, acceptability, and satisfaction with apps [15,16]. An analysis of user commentaries from women’s health apps indicates that, overall, women desire apps that are easy to use, contain new information, and are motivational [17]. Therefore, as consumers increasingly use mobile apps, health care providers, app developers, policy makers, and patients may benefit from a better understanding of the underlying factors that drive user demand and popularity of MIH apps.

The rapid proliferation of mHealth apps has not been accompanied by equal attention to understanding the factors that consumers prefer or the real-world usage patterns when selecting from a multitude of available apps [18,19]. Consumers have little reliable information to refer to when seeking apps for their health needs [18,19]. Furthermore, consumer advocacy groups and other professional organizations are largely unavailable to assess the quality of these apps, given the high number of apps available in app stores [20]. Considering an overall paucity of publicly available information pertaining to health apps, users generally make decisions pertaining to app use by considering easily available attributes such as title, price, star ratings, reviews, or downloads [21]. Existing research has indicated several factors involved in the process of app selection and download. Within the context of non–health-specific apps, consumers exhibit preferences for low-priced apps, in-app purchase options, and apps with recent updates as evidenced by higher user downloads [22]. Similarly, factors that relate to high user downloads of urology apps include expert involvement in app development, optional in-app purchases, low app cost, and high user ratings [23]. However, the literature on consumer preferences for MIH apps is still rather scarce. This necessitates a better understanding of user behavior within the context of intention to use and user satisfaction with these apps.

Considering the popularity of MIH apps, it is important to understand whether app characteristics (eg, price, ratings, or update age) indicated by previous studies remain influential within the context of perceived satisfaction and intent to use these apps. Therefore, the objective of this study was to examine the relationship between MIH app characteristics (app price, update age, app store, developer type, primary category/genre, content rating, in-app purchase, and in-app advertisement) and 2 outcomes, that is, end user’s perceived satisfaction (user ratings) and intent to use (downloads). Using app data from both the Apple App Store and Google Play Store, this study quantifies apps’ features and characteristics that may affect end users’ perceived satisfaction and intent to use. Given the specificity of MIH apps, this study also examined the influence of app developer type (ie, health care vs non–health care) on user behavior, that is, do users frequently download and rate apps developed by health care developers?

## Methods

### Source of Data

We measured the association between app characteristics, ratings, and downloads in a cross-sectional study of MIH apps available in the Apple App Store and Google Play Store. The dataset of MIH apps was built by scraping data from the Apple App Store [24] and Google Play Store [25] platforms using a Java-based scraper program called Node.js [26].

Scraping results returned apps in the same order as if the search was conducted by an end user. Only the first 200 app results for the Apple App Store and the first 250 app results (later reduced to 50 starting January 2017) for the Google Play Store were returned by the scraper program [27,28]. Therefore, the results of the scraping searches for this study contain apps that were higher ranked when searched and, therefore, most likely to be accessed by store visitors [23,29]. The Indiana University-Purdue University Indianapolis (IUPUI) institutional review board (IRB) approved and deemed this study as nonhuman subjects research.

### Search Strategy

We followed a 3-step process to identify a list of popular MIH apps focused on health education or decision-making support to pregnant women or parents/caregivers of infant. The data reflect app store content as of March 2017.

**Figure 1:**
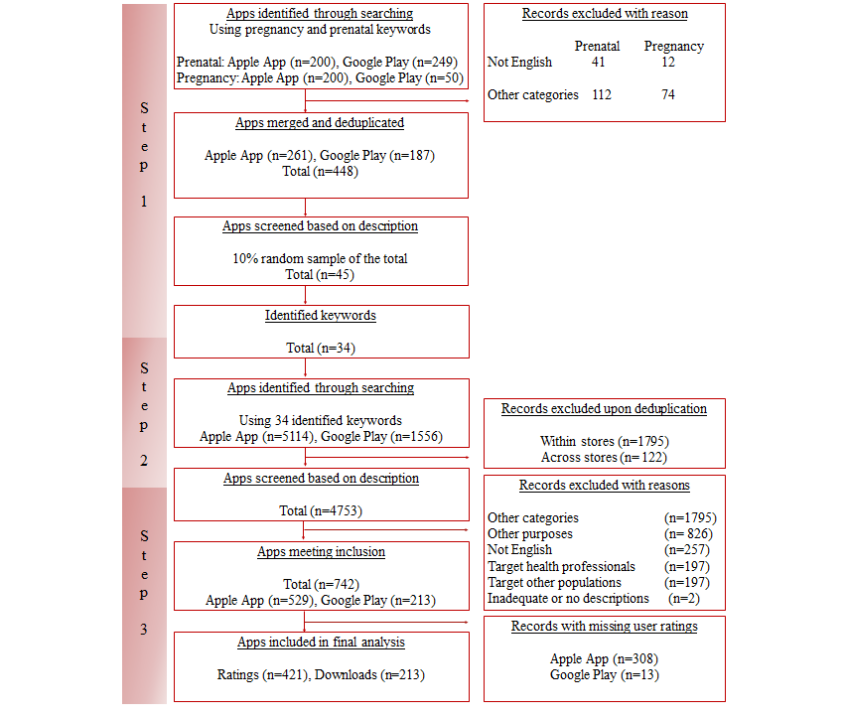
Flowchart detailing the sample selection process.

First, we identified a comprehensive list of relevant keywords that users might enter when searching for apps related to MIH. Search terms such as *pregnancy* and *prenatal* were used as the starting point for both the app stores, resulting in a total of 699 apps. From this, we examined app descriptions to identify apps that were in English language and belonged to education, health and fitness, and medical categories, which eliminated 34.0% of the apps. Subsequently, the app results from both stores were merged and duplicates were removed, thereby eliminating another 3.0% of apps (Figure 1). This resulted in a sample of 448 unique apps from the 2 stores (261 apps from the Apple App Store and 187 apps from the Google Play Store). From the resulting apps, we selected a simple random sample of 45 apps (45/448, 10.0%) and identified 34 additional keywords related to MIH from the app descriptions (Table 1).

Next, each of the 34 keywords was entered individually into a separate search to obtain a comprehensive set of apps for potential inclusion in the study. This resulted in a total sample of 6670 apps. The resultant apps were merged and deduplicated first within stores and then across stores for a total of 4753 unique apps in the dataset (Figure 1). If an app was available on both platforms, the Google Play Store version was included for analysis because the Google Play Store provides additional metadata, such as user downloads and in-app purchase option, which are not provided by the Apple App Store [30].

**Table 1:** List of keywords (N=34).

Keywords	Frequency of use within app description
Pregnant/pregnancy	170
Prenatal	63
Baby/babies	56
Child/children/childhood	29
Parents/parenting/parenthood	22
Birth	14
Mom/mum	12
Labor/delivery	8
Fetus/fetal	6
Maternal/maternity	5
Breastfeeding	4
Mother/motherhood	4
Infant	3
Obstetrics	3
Antenatal	3
Conception	3
Postnatal/postpartum	2
Gestation/gestational	2
Newborn	1
Intrapartum	1
Lactation	1

### App Selection

Overall, 2 reviewers (RB and CH) independently screened the app descriptions of all retrieved apps (n=4753) for inclusion and exclusion. Disagreements were resolved through discussion and consensus. Inclusion criteria were as follows: (1) description written in English language; (2) target users judged to be pregnant women, to-be parents, and other caregivers of infant children (ie, 0-1 year old as defined by Centers for Disease Control and Prevention, 2017) [31]; (3) listed in the medical, health and fitness, books and reference, or education categories in the Apple App Store or listed in the medical, health and fitness, books and reference, education, or parenting categories in the Google Play Store; and (4) described as intending to provide health education or user decision-making support. Exclusion criteria were as follows: (1) target users judged as health professionals, providers, or students in health professions as primary users; (2) had inadequate or no description provided; (3) apps meant to be used by members or people associated with special programs or health care facilities (eg, a clinic or hospital); (4) solely calculated gestational age and/or due date; and (5) solely used to identify baby names. The detailed review of app descriptions based on the inclusion and exclusion criteria resulted in a total of 742 apps (Figure 1).

### Data Extraction

For each included app, the data extracted included (1) average user rating (ie, *stars* 1 to 5), which reflects end users’ perceived satisfaction; (2) number of downloads, which measures intention to use; (3) app store; (4) prices in US dollars; (5) app developer type (health care and non–health care/unknown); (6) days since last app update; (7) primary categories/genre (medical, health and fitness, and other); (8) content rating (age restricted, not age restricted, and unrated); (9) in-app purchase option (yes/no); and (10) in-app advertisement presence (yes/no). The variables number of downloads, in-app purchase option, and in-app advertisement presence were available from the Google Play Store apps only.

### Data Analysis

The first outcome variable, average user ratings, was standardized as z-scores for the purpose of analysis. The Google Play Store offers continuous values to one-tenth of a point, whereas the Apple App Store rounds it to the nearest half point. To maintain consistency across stores, we converted it to a standardized z-score. Critically, the Apple App Store requires a minimum number of reviews before releasing average user ratings (ie, small numbers are suppressed), and the Google Play Store does not report ratings for unreviewed apps. Of the 742 apps in the sample, 43.3% of apps had no or suppressed user ratings. Therefore, these were all coded as missing values (n=321) and omitted from the analysis; hence, the analysis of user ratings reflects 421 apps from both stores (Figure 1). The second outcome variable, number of downloads, was available from the Google Play Store only; hence, the analysis of user downloads consists of 213 apps from 1 store. Download numbers could only be extracted as 1 of the 12 numeric range categories. For analysis, they were collapsed into 4 categories (1-500, 501-5000, 5001-50,000, and 50,001-50,000,000).

To categorize app developer type, a manual review of developer website provided by the app stores was conducted by the primary reviewer (RB). On the basis of the description provided, developers were categorized as a health care developer if they were identified as one of the following: government agency, US hospital system, US academic medical institution, medical specialty society, nonprofit health care organization, consumer organization with health focus, US physician, third-party payer, and pharmaceutical and medical technology companies [32]. Alternatively, developers were categorized as non–health care/unknown, based on the description provided, if they were not classified into 1 of the abovementioned categories or in cases where the website was not provided. The app update age was based on the number of days since the new version was released. This was calculated by subtracting the date of the last update from the date of data extraction, March 31, 2017. Only apps belonging to health and fitness, medical, books and reference, education, and parenting genres were included in this study. Owing to small sample sizes within some categories, combined apps belonging to books and reference, education, and parenting genre were pooled into a single category (other). Content rating was classified into 3 categories: not age restricted, age restricted, and unrated. The Apple App Store apps with ratings of 4+ were categorized as not age restricted; 9+, 12+, and 17+ as age restricted; and with no rating as unrated [33]. Similarly, the Google Play Store apps with ratings of everyone were categorized as not age restricted; low, medium, and high maturity as age restricted; and with no rating as unrated [34].

First, descriptive statistics were calculated and assessed. Next, the relationship between app characteristics and end users’ perceived satisfaction (user ratings) and intent to use (downloads) were examined in 2 separate regressions models. First, a multivariable linear regression assessed the relationship between app characteristics (app price, update age, app store, developer type, genre, and content rating) and standardized user ratings controlling for all other available app characteristics for both the Apple App Store and Google Play Store apps. Second, the association between app characteristics (standardized user rating, app price, update age, developer type, genre, in-app purchase, and in-app advertisement) and the number of app downloads was modeled using a series of ordinal logistic regressions for the Google Play Store apps only. Given the small sample size, the analysis of downloads could not be examined with all independent variables in a single model. Therefore, 6 models holding user ratings and price as constant with an additional independent variable were run. Statistical significance was assessed at the *P*<.05 level.

## Results

### App Characteristics

From the total of 421 apps that were included, 322 (75.5%) were free. Of the paid apps, the prices ranged from US $0.99 to US $10.92, with an average price of US $3.14 and a median of US $2.99. The number of days since the last update varied from 14 to 2888 (average 582 days). Only 102 (102/421, 24.2%) apps were developed by health care organizations. The average user rating was 3.79 out of 5. Furthermore, the modal category for user downloads was greater than 50,000, with 66 (66/213, 31.0%) apps (Table 2). In addition, from the 108 apps that offer in-app advertisements, as high as 104 (104/108, 96.3%) apps were offered free of cost to users, and for those that were paid, the prices ranged from US $0.99 to US $2.99.

**Table 2:** Descriptive statistics for independent and dependent variables.

Variables			Values
**Apps included in user rating analysis (N=421)**			
	Average user ratings (number of stars out of 5), mean (SD)		3.79 (0.98)
	App price (paid apps), mean (SD)		3.14 (2.13)
	Update age (days), mean (SD)		582 (624.44)
	**App store, n (%)**		
		Apple App Store	221 (52.5)
		Google Play Store	200 (47.5)
	**Developer type, n (%)**		
		Non–health care	319 (75.8)
		Health care	102 (24.2)
	**Primary category/genre, n (%)**		
		Health and fitness	225 (53.4)
		Medical	156 (37.1)
		Other (books and reference, education, and parenting)	40 (9.5)
	**Content rating, n (%)**		
		Not age restricted	319 (75.8)
		Age restricted	90 (21.3)
		Unrated	12 (2.9)
**Apps included in user download analysis (N=213), n (%)**			
	**Downloads**		
		1-500	43 (20.2)
		501-5000	52 (24.4)
		5001-50,000	52 (24.4)
		50,001-50,000,000	66 (31.0)
	**In-app purchase**		
		Yes	39 (18.3)
		No	174 (81.7)
	**In-app advertisement**		
		Yes	108 (50.7)
		No	105 (49.3)

### App Characteristics Associated With User Ratings

Compared with the Apple App Store, apps from the Google Play Store had, on average, 0.33 higher star ratings (*P*=.005; Table 3). Compared with *other* category, apps listed under the health and fitness genre had, on average, 0.41 lower star ratings (*P*=.01). Other factors negatively associated with satisfaction included older apps (ie, increasing app age; beta=−.0004; *P≤*.001) and apps with no age restriction (beta=−.32; *P*=.01). After controlling for other factors, developer type did not show statistically significant associations with rating (beta=−.20; *P*=.06).

**Table 3:** Multivariable linear regression for factors associated with standardized user ratings (N=421).

Variables		Estimates	SE	*P* value
Developer type health care^a^		−0.20	0.11	.06
Google Play platform^a^		0.33	0.12	.005
**Genre**				
	Other (books and reference, education, and parenting)	Ref^b^	Ref	Ref
	Medical	−0.19	0.17	.23
	Health and fitness	−0.41	0.17	.01
Update age		−0.0004	0.00008	<.001
**Content rating**				
	Age restricted apps	Ref	Ref	Ref
	Not age restricted apps	−0.32	0.13	.01
	Unrated apps	−0.51	0.32	.10
Price (US $)		0.03	0.03	.35

^a^The reference level for platform iOS and for developer type not health care developer.

^b^Ref: reference.

### App Characteristics Associated With User Downloads

Factors positively associated with user downloads were standardized user ratings (beta=.80; *P*<.001), in-app purchases (beta=1.12; *P*=.002), and in-app advertisement (beta=.64; *P*=.02) (Table 4). Compared with *other* category, apps listed under the medical genre had, on average, 1.63 lower star ratings (*P*<.001), and apps listed under health and fitness genre had, on average, 1.29 lower star ratings (*P*=.002). Other factors negatively associated with user downloads included price (beta=−.45; *P*=.003) and older apps (ie, increasing app age; beta=−.0008; *P*=.009). After controlling for other factors, developer type did not show statistically significant associations with downloads (beta=−.14; *P*=.63).

**Table 4:** Ordinal logistic regression for factors associated with user downloads (n=213).

Variables		Estimates	SE	*P* value
Standardized user rating		0.80	0.20	<.001
Price (US $)		−0.45	0.15	.003
Developer type health care		−0.14	0.30	.63
**Genre**				
	Other (books and reference, education, and parenting)	Ref^a^	Ref	Ref
	Medical	−1.63	0.46	<.001
	Health and fitness	−1.29	0.42	.002
Update age		−0.0008	0.0003	.009
In-app purchase		1.12	0.36	.002
In-app advertisement		0.64	0.27	.02

^a^Ref: reference.

## Discussion

### Principal Findings

This study uses publicly available open-source data to assess the factors related to user ratings (perceived satisfaction) and user downloads (intent to use) for MIH apps. To our knowledge, this is the first study that quantifies app features and characteristics that relate to user ratings and downloads for MIH apps using data from the Apple App Store and Google Play Store.

The Apple App Store and Google Play Store contain hundreds of apps related to MIH, many of which have been downloaded hundreds and thousands of times. Our findings suggest that price, user ratings, in-app purchase options, and presence of in-app advertisements were impactful predictors of user downloads. For instance, less expensive apps and apps with optional in-app purchases were associated with higher user downloads. Consumers tend to prefer apps that are free or of low cost with an ability to purchase additional features or functionalities via in-app purchases, as opposed to paying a higher price upfront [21,35]. Further examination on the quality of low-priced or free MIH apps is, therefore, needed.

Furthermore, the number of user downloads also increased with average user ratings, which suggests that perceived satisfaction with these apps is an important indicator related to new user preferences. This corroborates previous findings that most users tend to download apps with high user ratings [21,23]. Overall, consumers value Web-based word of mouth, thereby having a strong association with app sales and rankings [21,36]. However, high user ratings do not equate to quality, which is evident from an inaccurate instant blood pressure measurement app available in iTunes receiving high user ratings and positive user reviews [20].

In terms of genre, our findings suggest that apps in the health and fitness category have lower ratings and downloads, whereas apps in the medical category have fewer downloads. However, we cannot ascertain the exact reason behind why users may prefer MIH apps within specific categories over others, thereby calling for further investigation.

In addition, our results reveal that the availability of updates (ie, when was the app last updated) positively influences both user ratings and downloads. This is because updates act as a proxy of the app’s evolution [23]. Further, the presence of in-app advertisements is positively associated with user downloads. Although this finding may seem counterintuitive to the popular belief that in-app advertisements may cause annoyance and distraction to the user, it may, however, provide app developers with incentives to lower their app cost [23]. Our data show that from the apps that offer in-app advertisements, a vast majority of the apps were offered for free or for very low cost to users. Furthermore, unlike a previous report [23], our findings show that apps developed by health care developers are neither associated with higher ratings nor downloads.

These results may provide some correlational information to app developers, including health care organizations, about the types of apps that people tend to download and rate higher.

### Implications of Findings

Our findings could be applied to improve app design mechanisms that are currently in place for the MIH app market. Considering the sensitivity of MIH, we recommend that developers employ ways to increase health expert involvement in app design and content delivery.

A large majority (75.8%) of MIH apps included in this study were developed by non–health care organizations. This is consistent with previous reports on limited or nonexistent health expert involvement in app development within other health domains such as urology [23]. Prior studies that focus on evaluating the quality of mHealth apps have indicated missed opportunities pertaining to the timeliness and validity of the information that is being presented [6,37]. For example, out of 218 apps for the prevention of unintended pregnancy, approximately 40% of apps do not mention modern contraceptives, and from the remaining 60% of apps, less than 50% provide information on how to use them [28]. Similarly, from a sample of 10 free maternal and child health apps, only 4 apps provided information from evidence-based medical content [38].

Although these concerns have garnered attention from public agencies such as the US Food and Drug Administration (FDA), presently, the FDA only regulates apps that act as medical devices [39]. This calls for greater participation of health care organizations and other medical societies in app development, content review, and peer review process to increase app safety and accuracy [23]. It may also be beneficial for health care organizations and experts to review and *certify* health apps, similar to existing Web certification, such as the Health on the Net Foundation Code of Conduct, where the reliability and integrity of health information are evaluated against established standards [29]. Our results show no differences in user downloads between health care and non–health care organizations. Therefore, if health care organizations, in fact, provide more credible information, fewer consumers may receive this information. Hence, health care providers, app developers, and policy makers may consider strategies to review and promote apps to consumers based on information accuracy and trustworthiness.

### Limitations and Future Directions

In this study, we examined MIH apps from only 2 app stores, and information available in these app stores and developers’ websites were collected. However, the app stores and developers’ websites remain the main source of information available to consumers too. Thus, the study uses information similar to what would normally be available to consumers in a *real-world* context before downloading an app. Furthermore, each app store limits the number of search results that are returned on scraping data from the app stores. Next, unlike the Google Play Store, the Apple App Store does not provide data on the number of downloads; hence, only apps from the Google Play Store were included to assess the factors related to user downloads. In addition, apps that were included in the user rating analysis were somewhat different from apps that were excluded. Specifically, apps that were included versus excluded from the user rating analysis differed in the distribution of developer type and content rating. No significant differences were observed on other app characteristics (app price, update age, and primary genre) between included and excluded apps. In addition, questionnaires are often used to assess users’ intent to use an app. Positively, downloads are an objective measure that suggests intent to use [16,21], although we cannot state with certainty that downloaded apps may result in actual use. While categorizing the app developer type, we used the classification system based on the description provided on the developer website. It is possible that some app developers that were classified under non–health care developers may have consulted medical experts during app design. In addition, for app developers that were classified as health care developers, there was no known way to quantify the level of involvement by medical experts.

We suggest future studies focus on establishing consistent guidelines for the disclosure of health care professional’s participation and measures to quantify it. We also recommend future studies apply the same approach to other health topics and compare their results with this study. Although we found associations of app characteristics with perceived satisfaction and intent to use, we were not able to identify their impact on learning or behavior change because of app use. Therefore, we recommend future research and inquiry to focus on collecting data from users pertaining to their learning and behavior impact from app use. At present, we lack a standardized format/clinical guideline for the evaluation of accuracy of clinical content or included topics within apps, which necessitates further study and recommendations in this area.

### Conclusions

A large majority of MIH apps were developed by non–health care organizations, which raises concern about the clinical accuracy and quality of MIH app content. No differences in ratings or downloads were observed between health care and non–health care organizations. Therefore, if health care organizations, in fact, provide more credible information, fewer consumers may receive this information. Health care providers, app developers, and policy makers may consider strategies to review and promote evidence-based and trustworthy apps to consumers.

mHealth apps are increasingly becoming popular and can be used as a tool for MIH care delivery. However, the design and delivery of effective MIH apps still remain a challenging issue. Considering the lack of standard guidelines for app development, or selection, users typically consider publicly available app characteristics to make decisions pertaining to app use and satisfaction. Therefore, we examined the relationship between app characteristics, perceived satisfaction, and intent to use by using cross-sectional data from 2 app stores. We observed that app price, update age, user ratings, in-app purchases, and in-app advertisements are important predictors for intent to use, whereas update age is an important indicator for perceived satisfaction. Most importantly, our findings revealed that apps developed by health care developers were neither associated with higher perceived satisfaction nor intent to use. Knowledge of factors related to ratings and downloads may benefit app developers and help inform future marketing and development strategies.

## Abbreviations

FDA: Food and Drug Administration
IRB: institutional review board
IUPUI: Indiana University-Purdue University Indianapolis
MIH: maternal and infant health
mHealth: mobile health
